# Over-Expression of the *Pikh* Gene with a *CaMV 35S* Promoter Leads to Improved Blast Disease (*Magnaporthe oryzae*) Tolerance in Rice

**DOI:** 10.3389/fpls.2016.00773

**Published:** 2016-06-17

**Authors:** Parisa Azizi, Mohd Y. Rafii, Siti N. A. Abdullah, Mohamed M. Hanafi, M. Maziah, Mahbod Sahebi, Sadegh Ashkani, Sima Taheri, Mohammad F. Jahromi

**Affiliations:** ^1^Laboratory of Food Crops, Institute of Tropical Agriculture, Universiti Putra MalaysiaSerdang, Malaysia; ^2^Laboratory of Plantation Crop, Institute of Tropical Agriculture, Universiti Putra MalaysiaSerdang, Malaysia; ^3^Department of Biochemistry, Faculty of Biotechnology and Biomolecular Science, Universiti Putra MalaysiaSerdang, Malaysia; ^4^Department of Agronomy and Plant Breeding, Shahr-e-Rey Branch, Islamic Azad UniversityTehran, Iran; ^5^Depatment of Crop Science, Faculty of Agriculture, Universiti Putra MalaysiaSerdang, Malaysia; ^6^Laboratory of animal production, Institute of Tropical Agriculture, Universiti Putra MalaysiaSerdang, Malaysia

**Keywords:** plant pathogen, coding DNA sequence, *Agrobacterium*-mediated transformation, real-time quantitative PCR, high performance liquid chromatography

## Abstract

*Magnaporthe oryzae* is a rice blast fungus and plant pathogen that causes a serious rice disease and, therefore, poses a threat to the world's second most important food security crop. Plant transformation technology has become an adaptable system for cultivar improvement and to functionally analyze genes in plants. The objective of this study was to determine the effects (through over-expressing and using the CaMV 35S promoter) of *Pikh* on MR219 resistance because it is a rice variety that is susceptible to the blast fungus pathotype P7.2. Thus, a full DNA and coding DNA sequence (CDS) of the *Pikh* gene, 3172 bp, and 1206 bp in length, were obtained through amplifying the gDNA and cDNA template from a PH9-resistant rice variety using a specific primer. *Agrobacterium*-mediated transformation technology was also used to introduce the *Pikh* gene into the MR219 callus. Subsequently, transgenic plants were evaluated from the DNA to protein stages using polymerase chain reaction (PCR), semi-quantitative RT-PCR, real-time quantitative PCR and high performance liquid chromatography (HPLC). Transgenic plants were also compared with a control using a real-time quantification technique (to quantify the pathogen population), and transgenic and control plants were challenged with the local most virulent *M. oryzae* pathotype, P7.2. Based on the results, the *Pikh* gene encodes a hydrophilic protein with 18 sheets, 4 helixes, and 21 coils. This protein contains 401 amino acids, among which the amino acid sequence from 1 to 376 is a non-cytoplasmic region, that from 377 to 397 is a transmembrane region, and that from 398 to 401 is a cytoplasmic region with no identified disordered regions. The *Pikh* gene was up-regulated in the transgenic plants compared with the control plants. The quantity of the amino acid leucine in the transgenic rice plants increased significantly from 17.131 in the wild-type to 47.865 mg g^−1^ in transgenic plants. The *M. oryzae* population was constant at 31, 48, and 72 h after inoculation in transgenic plants, while it was increased in the inoculated control plants. This study successfully clarified that over-expression of the *Pikh* gene in transgenic plants can improve their blast resistance against the *M. oryzae* pathotype P7.2.

## Introduction

Over the past few decades, using resistance genes in resistant rice cultivars has been more desirable among different blast disease control strategies (Ashkani et al., 2015). Thus, the demand for rice breeders to produce durably resistant rice varieties is high. Bio-engineering techniques, such as over-expression and RNA silencing, could efficiently improve rice tolerance to fungal diseases (Kanzaki et al., 2002; Li et al., 2012, 2014). Currently, ~100 blast resistance genes have been identified and mapped in various rice genotypes (4% are from wild species, 45% are from Japonica, and 51% are from Indica cultivars), while only 19 of these genes have been cloned and characterized (Sharma et al., 2012). Most of these blast resistance genes belong to the nucleotide-binding site-leucine rich repeat (NBS-LRR) class of genes (Sharma et al., 2012). These genes can be applied with breeding and genetic engineering programs for introgressing a high degree of tolerance into well-performing commercial cultivars with susceptibility to blast disease (Zhang, 2007). This broad-spectrum blast-resistance gene introgression has been reported from *O. rufipogon* in Indica rice cultivars (Ram et al., 2007). Gene cloning is an essential step in understanding resistance gene structure and functions (Alberts et al., 2002). The breakthrough in blast resistance gene cloning came with *Pib* cloning in Japan in 1999 ~90 years after blast genetics was first studied (Wang et al., 1999). Thereafter, *Pita*, which is another important blast resistance gene, was cloned in 2000 by American researchers (Bryan et al., 2000). *Pikh* (*Pi54*) was cloned from an Indica cultivar (Tetep) in India after a 5-year gap (Sharma et al., 2005a). The *Pikh* gene was successfully assayed using functional studies after its identification (Sharma et al., 2005b). Transgenic lines containing the *Pikh* were established to confer a high degree of resistance to various *M. oryzae* isolates (Rai et al., 2011). *Pikh* gene has been wrongly reported as a member of NBS-LRR gene family (Sharma et al., 2012), while we clearly identified it as LRR type of R protein. *Agrobacterium-*mediated transformation is a desired method for a wide range of plant modifications (Tzfira and Citovsky, 2006).

Different types of promoters have been used in biotechnology applications based on the intended type of gene expression control, including constitutive (active in most of the tissues and stages), tissue-specific or development-stage-specific, inducible (regulated by external stimuli) and synthetic promoters (Hernandez-Garcia and Finer, 2014). Constitutive promoters, such as CaMV 35S, are typically applied when assessing transgenes because transgene specific effects may be easier to score if the transgene can be expressed in various tissues under diverse conditions (Benfey and Chua, 1990). A direct strategy for manipulating enhanced normal tolerance using constitutive expression of naturally inducible defenses has been suggested; however, activation of defense gene transcription triggers the expression of various inducible defense mechanisms (Zhu et al., 1994). The objective of this study was to determine the effects (through over-expression) of *Pikh* on MR219 resistance as a rice variety that is susceptible to blast fungal pathotype P7.2.

## Materials and methods

### Plant materials and inoculation

The Malaysian rice varieties MR219 (Highly susceptible) and PH9 (most resistant variety; Azizi et al., 2015a) as well as an *M. oryzae* strain (P7.2) were provided by the Malaysian Agricultural Research and Development Institute (MARDI). Rice seeds were soaked in water for 3 days and germinated on moist Whatman filter paper in Petri dishes. Subsequently, the seeds were planted in pots using autoclaved potting soil, transferred to a glasshouse and maintained at 25–30°C for 3 weeks. Fifteen MR219 and PH9 rice seedlings from the three replicates at three leaf stage were sprayed and inoculated by one liter of a *M. oryzae* spore suspension containing 3 × 10^5^ spores/mL. The leaves were collected after 31 h of inoculation and served as plant material in this study. During all PCRs steps, negative controls were run but, they are not shown in the gel figures.

### Identifying the rice blast resistance gene, *Pikh*, in the PH9 resistant rice variety

Genomic DNA (gDNA) was extracted from leaves of PH9 resistant rice variety after inoculation by *M. oryzae* using Doyle method (Doyle, 1990). The full length of *Pikh* gene was achieved using following primers (design based on the GenBank reference GU258499.1) and PCR (Taq DNA Polymerase, Vivantis, Chino, California, USA) program: 94°C for 2 min and 35 cycles of 94°C for 30 s, 58°C for 30 s, and 72°C for 60 s. The PCR program concluded with a final extension step for 7 min at 72°C. The PCR products were separated on a 1.5% agarose gel stained with ethidium bromide. The expected band (~3172 bp) was purified using a QIAquick® gel extraction kit (QIAGEN, Hilden, North Rhine Westphalia, Germany) and sent for sequencing.

Forward primer: 5′-ATG CTCTTGGTCCTTTCTATCTTGTC-3′
Reverse primer: 5′-CAT GAGAACATATGTAAGCTTGTGC-3′

### Isolating full coding DNA sequence (CDs) of *Pikh*, and construction of an over-expression vector

#### RNA extraction and semi-quantitative RT-PCR analysis

The total RNA was extracted from treated leaves of the *M. oryzae* PH9 variety using the TRIzol method (Simms et al., 1993). Reverse transcriptase-PCR was carried out to isolate the *Pikh* gene from the PH9 variety. One microgram per microliter of total RNA was transcribed to the first-strand cDNA fragments using Super Script III (Invitrogen, Carlsbad, California, USA). The reactions were incubated at 50°C for 60 min and heated for inactivation at 70°C for 15 min. Next, the template cDNA was amplified using the following *Pikh* specific primers designed in accordance with the cDNA sequence homologies (GenBank: GU258499.1) using Primer Premier 5.0 software (Ren et al., 2004).

Forward primer: 5′-CTA GTTCAATTGCTTTAAG-3′
Reverse primer: 5′-ATG AGTAAAATGAAGAAGC-3′

The following PCR (Taq DNA Polymerase, Vivantis, Chino, California, USA) program was used: 94°C for 2 min and 35 cycles of 94°C for 30 s, 55°C for 30 s, and 72°C for 30 s. The PCR program concluded with a final extension step for 7 min at 72°C. The PCR products were separated on a 1.5% agarose gel, which was stained with ethidium bromide. The expected band (~1206 bp) was purified using a QIAquick® gel extraction kit (QIAGEN, Hilden, North Rhine Westphalia, Germany) and sent for sequencing.

### Constructing recombinant plasmids

The purified PCR product (1206 bp) was directly inserted into the pDriveU/A cloning vector (QIAGEN, Hilden, North Rhine Westphalia, Germany) using a PCR Cloning^plus^ Kit (QIAGEN, Hilden, North Rhine Westphalia, Germany). The ligated pDrive vectors were then transformed into *E. coli* EZ cells and cultured overnight (16 h, 37°C) in LB agar medium containing X-gal, IPTG, and ampicillin.

### Identification of recombinant plasmids

- Plasmid purification
  Approximately 30 positive clones were selected randomly, 8 positive colonies were subjected to colony PCR using M13 primers (forward and reverse), and the PCR protocol was carried out using a Taq DNA polymerase kit (Fermentas, Waltham, Massachusetts, USA). The plasmids were then purified using a plasmid purification kit (QIAGEN, Hilden, North Rhine Westphalia, Germany).
- Confirmation of recombinant clones through digesting recombinant plasmids and DNA sequencing
  When the above procedure was complete, 15 μL (at 63 ng/ μL concentration) of the pure vector with the desired genetic code was sent for sequencing. Up to 1 μg plasmid DNA extracted from each positive clone was placed in a 0.5 mL micro centrifuge tube; 2 μL of buffer 10X and sterile H_2_O was added to a final volume of 19 μL and mixed through pipetting and vortexing; 1 μL of the restriction enzyme *EcoR I* was then added (Fermentas, Waltham, Massachusetts, USA), and the sample was incubated at 37°C for at least 1 h.

### Constructing an expression clone

Gateway® Technology with Clonase™ II (Invitrogen, Carlsbad, California, USA) was used to construct the expression clone.

### Constructing entry clones

The KAPA HiFi Hot Start DNA polymerase kit was used to generate attB-PCR products using following primers designed based on the structure of the pDONOR/Zeo vector and the manufacturer's Gateway technology.

Forward *Pikh*-anchor: 5′-GGG GACAAGTTTGTACAAAAAAGCAGGCTTC CTAGTTCAATTGCTTTAAG-3′
Reverse *Pikh*-anchor: 5′-GGG GACCACTTTGTACAAGAAAGCTGGGTC ATGAGTAAAATGAAGAAGC-3′

The QIAquick® gel extraction kit (QIAGEN, Hilden, North Rhine Westphalia, Germany) was used to the target gene (1267 bp) from the gel electrophoresis results. In the next step, the attB-PCR product was cloned, and recombinant plasmids were purified using the QIAprep® Spin Miniprep Kit (QIAGEN, Hilden, North Rhine Westphalia, Germany) to construct expression clones.

### Constructing expression clones

The LR and BP recombination reaction procedures were performed in accordance with the manufacturer's procedures for the Gateway® Technology kit (Invitrogen, Carlsbad, California, USA).

### Transforming competent cells

The *E. coli* TOP10 strain (QIAGEN, Hilden, North Rhine Westphalia, Germany) was used as competent cells for transformation because it lacks a ccdA gene in its structure; therefore, it does not prevent negative selection through the ccdB gene of the pDONOR™/Zeo vector.

### Verifying the recombinant entry and expression clones through colony PCR and sequencing

**Figure 1:**
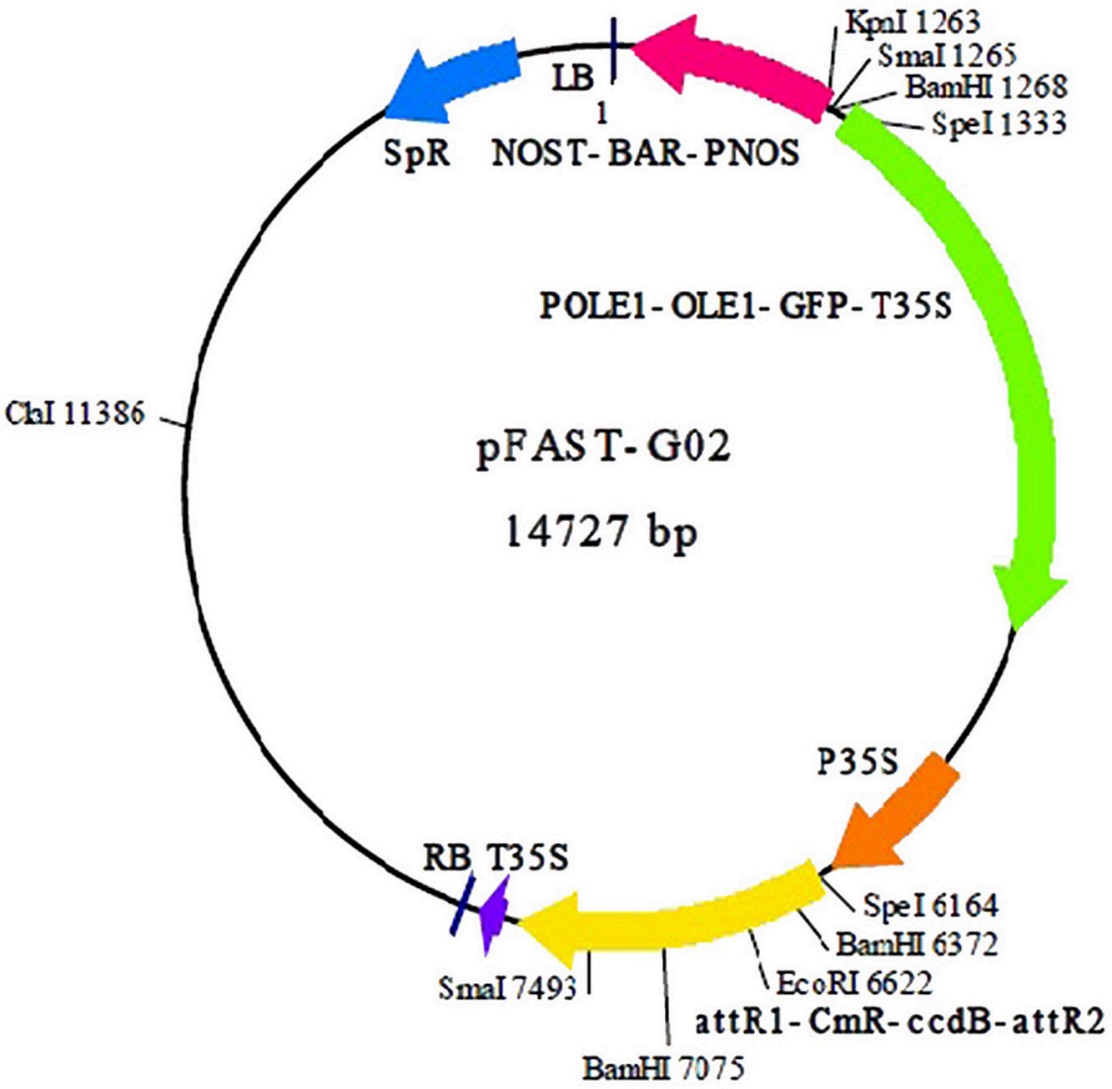
**Plasmid map of pFAST-G02 used to construct the expression clone (Shimada et al., 2010)**.

Sixteen single colonies from two groups (recombinant entry and expression clones) were cultured overnight and randomly selected for amplification using a Taq DNA polymerase kit (Fermentas, Waltham, Massachusetts, USA). The plasmid purification kit (QIAGEN, Hilden, North Rhine Westphalia, Germany), was used to purify recombinant entry and expression clones (Figure 1) using manufacturer's procedures. The purified plasmids then were sent for sequencing.

### Transformation procedure

Ten sterile MR219 seeds were placed in each Petri dish (100 × 15 mm) containing 20 mL MS-2,4-D medium (MS salts and vitamins, 300 mg/L casamino acid, 2 mg/L 2,4-D, and 2.8 g/L Gelrite, pH 5.8). Three Petri dishes were measured as replicates, and nine petri dishes in total (3 replicates) were analyzed. Next, the seed cultures were incubated in dark conditions at 25°C. After 3 weeks, yellowish white embryogenic calli developed on the scutellar surface. *Agrobacterium*-mediated transformation (Strain LBA4404) was used to transfer the expression clone to the MR219 calli. The *Agrobacterium* strain LBA 4404 containing an expression vector was streaked on an LB solid medium containing both streptomycin (100 μg/mL; antibiotic appropriate for vector used) and rifampicin (50 μg/mL; bacterial selective agents). The cultured plates were incubated in dark conditions for 2 days. *Agrobacterium-*containing expression vectors were transformed into the MR219 embryogenic calli using the Datta and Datta (2006) protocol. Next, transformed MR219 embryogenic calli were regenerated using the procedure explained in Table 1.

**Table 1:** **Different stages of the tissue culture procedure used to regenerate transgenic plantlets from transformed embryogenic calli (Datta and Datta, 2006; Azizi et al., 2015b)**.

**Stages of tissue culture protocol**	**Medium**	**Media components**	**Incubation conditions**
Production of embryogenic calli	MS-2,4-D	MS salts and vitamins, 300 mg/L casamino acid, 2 mg/L 2,4-D, and 2.8 g/L gelrite, pH 5.8	Dark condition at 25°C for 3 weeks
Proliferation of transformed MR219 calli	MS-2,4-D + cefotoxime	MS salts and vitamins, 300 mg/L casamino acid, 2 mg/L 2,4-D, 250 mg/L Cefotoxime and 2.8 g/L gelrite, pH 5.8	Dark condition at 25°C for 10 days
Selection transformed calli (First selection media)	MS-2,4-D + cefotoxime + BASTA	MS salts and vitamins, 300 mg/L casamino acid, 2 mg/L 2,4-D, 250 mg/L cefotoxime, 3 mg/L herbicide phosphinothricin (BASTA) and 2.8 g/L gelrite, pH 5.8	2 weeks at 25°C under dark condition
Selection transformed calli (Second selection media)	MS-2,4-D + cefotoxime + BASTA	MS salts and vitamins, 300 mg/L casamino acid, 2 mg/L 2,4-D, 250 mg/L cefotoxime, 3 mg/L herbicide phosphinothricin (BASTA) and 2.8 g/L gelrite, pH 5.8	2 weeks at 25°C under dark condition
Selection transformed calli (Third selection media)	MS-2,4-D + cefotoxime + BASTA	MS salts and vitamins, 300 mg/L casamino acid, 2 mg/L 2,4-D, 250 mg/L cefotoxime, 3 mg/L herbicide phosphinothricin (BASTA) and 2.8 g/L gelrite, pH 5.8	2 weeks at 25°C under dark condition
Shoot production from surviving healthy embryogenic calli	MSKN regeneration medium	MS salts, MS vitamins, 2 mg/L kinetin, 1 mg/L NAA, 300 mg/L casamino acid, 50 mg/L cefotoxime, 30 g/L sucrose, 0.1 g/L myo-inositol, 2.8 g/L gelrite, pH 5.8	Dark for 20 days at 25°C
Regeneration of plantlets	Regeneration media (MSKN)	MS salts, MS vitamins, 2 mg/L kinetin, 1 mg/L NAA, 300 mg/L casamino acid, 30 g/L sucrose, 0.1 g/L myo-inositol, 2.8 g/L gelrite, pH 5.8	Light condition at 27°C with a 16-h photo period (110 μmol/m^2^/s) for 20 days
Root production	Rooting medium (MSO)	MS salts, MS vitamins, 300 mg/L casamino acid, 30 g/L sucrose, 0.1 g/L myo-inositol, 2.8 g/L gelrite, pH 5.8	Light condition at 27°C with a 16-h photo period (110 μmol/m^2^/s) for 20 days

When a well-developed root system was observed, culture medium was gently removed from the roots of the plantlets using water. Next, the plantlets were individually transferred to the Yoshida culture solution in a greenhouse with a 14 h photo period (160 μmol/m^2^/s), 95% relative humidity, and 29°C day/light temperature and maintained for 21 days. Subsequently, the plants with vigorous root systems were transferred into pots containing paddy field soil, water (1 L per pot and 500 mL each day until maturity), and sufficient initial fertilizer [a mixture of fertilizer 2.5 g of (NH_4_) SO_2_, 1.25 g of P_2_O_5_, 0.75 g of K_2_O per pot]. Approximately 2.5 g of (NH_4_) SO_2_was added into each pot at the initial flowering stage. Panicles were harvested when 85% of grains turned straw gold in color.

### GFP monitoring in the transgenic MR219 seeds

We monitored the GFP expressed in the transgenic seeds collected from the T_0_ and T_1_ generations using a florescence microscope (Leica MZFL111) prepared with a GFP2 filter adjusted to detect GFP expression in rice seeds. The Leica DC 200 system was set to the fluorescence microscope to collect images using the Leica DC Viewer software.

### Transgenic MR219 plant analysis

Transformed plants were maintained in the greenhouse, and the seeds were harvested upon full maturation. The 3G plant PCR kit (KAPA, Cape Town, Western Cape, South Africa), was used to analyze the putative transgenic plants. The presence of transgenic plants in the T_1_ lines was assessed based on PCR amplification using two sets of primers designed specifically for the *Pikh* gene and *CaMV35S* promoter sequences (Table 2). The PCR program was as follows: initial denaturation at 95°C for 3 min, 35 cycles of denaturation at 95°C for 20 s, annealing at 55°C (*Pikh*) and 58°C (*CaMV35S*) for 15 s, and extension at 72°C for 1 min; the final extension was 1 min at 72°C.

**Table 2:** **List of primers used to confirm the transgenic MR219 plant**.

**Primer**	**Forward 5′ → 3′**	**Reverse 5′ → 3′**	**Size of amplicon**
*Pikh*	CTAGTTCAATTGCTTTAAG	ATGAGTAAAATGAAGAAGC	1206 bp
*CaMV35S*	CCGACAGTGGTCCCAAAGAT	ATGAGTAAAATGAAGAAGC	1502 bp

### Reverse transcriptase-PCR analysis

Reverse transcriptase RT-PCR was used to study *Pikh* gene expression in the inoculated transgenic and untreated transgenic rice plants using SuperScript III (Invitrogen, Carlsbad, California, USA). The *18S rRNA* gene was used as an internal control and amplified using a specific primer (Table 3).

**Table 3:** **List of primers used for semi-quantitative RT-PCR of *Pikh* and *18S rRNA***.

**Primer**	**Forward 5′ → 3′**	**Reverse 5′ → 3′**	**Size of amplicon**
*Pikh*	CTAGTTCAATTGCTTTAAG	ATGAGTAAAATGAAGAAGC	1206 bp
*18S rRNA*	ATGATAACTCGACGGATCGC	CTTGGATGTGGTAGCCGTTT	168 bp

### Analyzing the *Pikh* gene expression in T_1_ transgenic plants using real-time quantitative PCR

Real-time qRT-PCR was used to evaluate the expression levels of the *Pikh* gene in the leaf tissue of the transgenic plants in response to *M. oryzae* (pathotype P7.2) inoculation after 31 h compared with the non-inoculated plants. All data were from three independent biological replicates. The *Pikh, 18S rRNA*, and α*-Tubulin* genes were amplified using specific primers (Table 4).

**Table 4:** **List of primers used for real-time quantitative PCR**.

**Gene**	**Forward 5′ → 3′**	**Reverse 5′ → 3′**	**Size of amplicon**
*Pikh*	AAGATTTTCGAGGCTCTTCTCTA	ATGAATCTGTTTCCTCGTCTTG	172 bp
*18SrRNA*	ATGATAACTCGACGGATCGC	CTTGGATGTGGTAGCCGTTT	168 bp
*α-Tubulin*	GGAAATACATGGCTTGCTGCTT	TCTCTTCGTCTTGATGGTTGCA	89 bp

18SrRNA and α-Tubulin were used as reference genes.

### Amino acid analysis of transgenic plants

The amino acid analysis was performed using the Waters HPLC system (E2696, USA) and 2475 fluorescence detector ACCQ-Fluor™ Reagent Kit (USA). The column featured a 3.9 inner diameter and was 150 mm long, ACCQ-Tag™ (Waters, USA). The mobile phase was as follows: (A) 10% ACCQ-Tag™ Eluent solution in water and (B) 60% acetonitrile in water. The flow rate was 1 mLmin^−1^. This portion of the study was performed using data from three independent biological replicates for both transgenic and wild-type MR219 rice plants, separately.

- Sample preparation
  One-hundred-and-fifty milligrams of freeze-dried plant was ground and poured into a glass tube, 5 mL of 6 N HCL was added, and the sample was incubated at 110°C for 24 h for hydrolysis. The day after hydrolysis, the sample was poured into a volumetric flask (100 mL), 4 mL of stock 2.5 mM AABA (α-amino butyric acid) was added, and sterile water was added for a final volume of 100 mL. An aliquot of the sample was filtered through a syringe filter. Ten microliters of the filtered sample was placed in a clean 1.5 mL micro centrifuge tube, and the AccQ Fluor reagent (70 μL of borate buffer, and 20 μL of AccQ reagent) was added to a final volume of 100 μL. The sample was maintained at RT for 1 min and incubated at 55°C for 10 min. Next, 5 μL of the sample was injected to an HPLC auto-sampler vial for an amino acid analysis. The concentration of each amino acid standard used to calibrate the HPLC is shown in Supplementary Table 1. In the procedure for amino acid analysis using HPLC, the internal standard is AABA, and the analytes are individual amino acids. In the first step, a standard solution was generated containing a known concentration of all amino acids and a known concentration of internal standard (Supplementary Table 1). For the known amino acid standards, we solved for F using the following equation.
  $$\begin{array}{l} \;\frac{\text{Area internal standard peak }(\text{AABA})\;}{\text{Area of amino acid standard peak }}\\ \;=\text{F}\frac{\text{Concentration of internal standard }(\text{AABA})}{\text{Concentration of amino acid standard}} \end{array}$$
  Note that in the equation above, we know everything but F, which is a dimensionless constant.
  In the next step, the solutions containing a known quantity of internal standard (AABA at 0.2 μg/mL) and unknown quantity of each amino acid were injected into HPLC. The concentration of the unknown amino acid can be calculated using the above equation.
- Statistical analysis of the HPLC results
  All data were analyzed using the general linear model (GLM) procedure from the SAS software for analysis of variance (Timm and Mieczkowski, 1997).

### Bioinformatic analysis

The hydrophobicity, hydrophilicity, and amino acid composition of the protein encoded by the *Pikh* gene were predicted online using ProtScale (http://web.expasy.org/protscale/) in the ExPASy tool kit. To determine the sub-cellular localization of the protein PSORT II Prediction (Psort, 1997), MEMSAT-SV, Cell-Ploc (Chou and Shen, 2007, 2008, 2010) and BacelLO (Pierleoni et al., 2006) software was used. Disordered regions of the protein were surveyed using DISOPRED 2 (Ward et al., 2004). The secondary structure predictions were carried out using the ITASSER (Zhang, 2008; Roy et al., 2010; Yang and Zhang, 2015; Yang et al., 2015) and PsiPred (McGuffin et al., 2000) programs. The protein 3D structure was predicted using ITASSER and Phyre2 (Kelley et al., 2015). Finally, functional domains in Pikh protein structure were predicted using GenBank KM501045.1 and corresponding protein AIY55350 and depending on the algorithm used at NCBI (Marchler-Bauer et al., 2009).

### Differentiation of the pathogen population between the inoculated transgenic and untransformed plants using a bacterial quantification technique

#### Real-time quantitative PCR assay

For the real-time quantitative PCR assay, the total DNA was extracted from the T_1_ transformed and wild type MR219 seedlings inoculated by the *M. oryzae* pathotype P7.2 at 3 × 10^5^ spores/mL 31, 48, and 72 h after inoculation using an i-genomic BYF DNA extraction mini kit (Intron Biotechnology, Seongnam, Gyeonggi-do, Korea).

The absolute quantification of the *M. oryzae* populations among the inoculated rice plants was carried out based on the standard curve method using real-time quantitative PCR. Standard curves were constructed using the number of copies for the *28S rDNA* gene plotted against the quantification cycle (Cq) obtained from 10-fold serial dilutions of PCR products from a pure fungal culture. To prepare the standard curves, we used a purified *28S rDNA* gene produced from a previous study (Azizi et al., 2015a).

The copy number of the *28S rDNA* per mL of elution buffer was calculated using the following formula [available online in the Genomics and Sequencing Center of University of Rhode Island (URI^1^); Azizi et al., 2015a].

Real-time quantitative PCR was performed using a BioRad CFX96 Real-time quantitative PCR system (BioRad, USA) with optical grade plates. The primers used to quantitate the different fungal populations are shown in Table 5. The real-time quantitative PCR reaction was performed with a total volume of 25 μL using the QIAGEN Quantifast SYBR Green PCR, USA. Each reaction consisted of 12.5 μl of 2 × SYBR Green Master Mix, 1 μL of 10 μM forward primer, 1 μL of 10 μM reverse primer, 2 μL of DNA sample and 8.5 μL of nuclease-free water. Each sample was assayed with three replications. A no-template control (NTC) was included in the real-time quantitative PCR amplification to rule out any cross-contamination. Real-time quantitative PCR cycling conditions comprised an initial 5 min at 95°C followed by 40 cycles of 10 s at 95°C and 30 s at 60°C. When the amplification was complete, the specificity of the amplified product was confirmed using a melting curve analysis. The real-time quantitative PCR products were incubated by raising the temperature from 70 to 95 in 0.5°C increments with a 5 s hold at each increment.

**Table 5:** **Primers used for the real-time quantitative PCR assay to target *M. oryzae***.

**Target group**		**Sequence 5′ → 3′**	**Size of amplicon**
*28S rDNA*	Forward	TACGAGAGGAACCGCTCATTCAGATAATTA	330 bp
	Reverse	TCAGCAGATCGTAACGATAAAGCTACTC	

### Resistance analysis and challenging T_1_ plants with the most virulence *M. oryzae* fungal pathotype P7.2 in Malaysia

The T_1_plants were evaluated in the glasshouse for the blast disease reaction to *M. oryzae* pathotype P7.2. Inoculation was performed at the seedling stage (3-week-old rice plants) using a 250 mL fungus spore suspension at 3 × 10^5^ spores/mL. The treated T_1_and untransformed (control) plants were covered with plastic bags to maintain the plants in high humidity and under dark conditions for 24 h. Next, the plastic bags were removed from the plants, which were maintained under normal conditions in the glasshouse. The inoculation experiments were repeated three times for all varieties. Three components of partial resistance, including blast lesion degree (BLD), blast lesion type (BLT), and percentage disease leaf area (%DLA), were measured and compared between T_1_and control plants 7 days after inoculation.

## Results

### Isolation of a full-length and coding region for the *Pikh* gene

The full-length *Pikh* and its transcript were obtained through amplifying the gDNA and cDNA template using gene-specific primers (Supplementary Figures 1A,B). The presence of a cloned gene in the plasmid was confirmed through digestion and sequencing (Supplementary Figure 1C). The complete *Pikh* gene and its CDS with 3172 and 1206 bp nucleotides encode a deduced protein of 401 amino acids, which was submitted to NCBI with the accession number (KM501045) and 100% identity with GU258499.1.

In the next step, expression clones were constructed using an attB-PCR product (Supplementary Figure 2A); subsequently, PCR colony and sequencing confirmed the presence of the *Pikh* gene in the BP and LR recombination reactions (Supplementary Figures 2B,C), respectively. Finally, the recombinant expression colonies were introduced into the *Agrobacterium* competent cells using a freeze-thaw technique. A PCR colony of the transformed *Agrobacterium* competent cells verified the transformation steps (Supplementary Figure 2D).

### Analysis of the T_1_ transgenic rice plants

**Figure 2:**
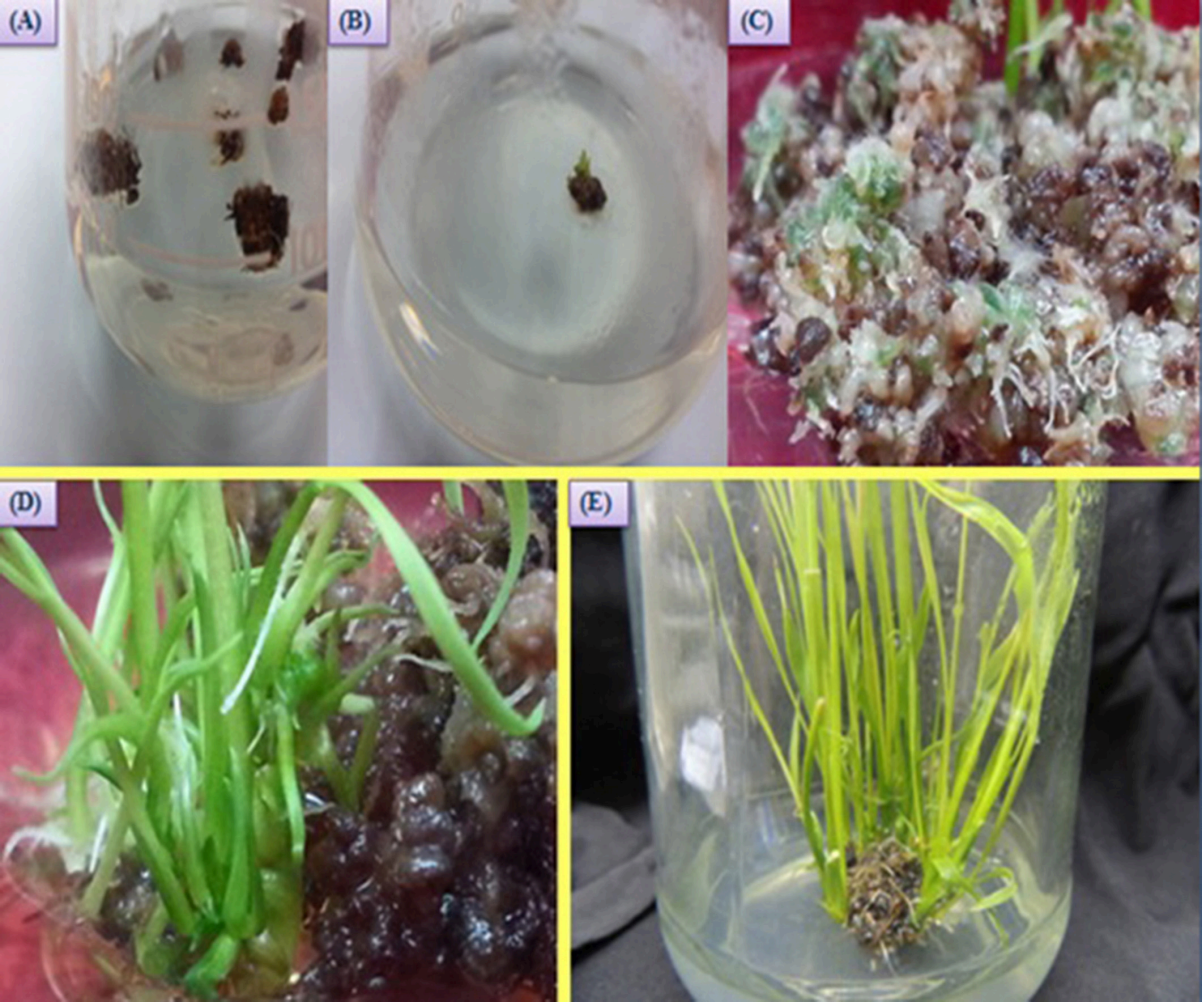
**Regeneration of T_0_ plantlets from transformed MR219 calli. (A)** Transformed calli on the selection media; **(B,C)** production of green spots on the calli cells, and **(D,E)** regenerated transgenic shoots of MR219 from calli.

Four and nine transgenic plants were identified at the end of the selection process as T_0_ and T_1_ transgenic generations, respectively (Figure 2). The regenerated plants continued to grow and remained green; however, the untransformed calli died after selection. However, the T_0_ plants were not strong as MR219 control plants. The results obtained from a PCR analysis of the T_1_transgenic plants yielded two bands at 1206 and 1507 bp (Supplementary Figures 3A,B).

### GFP monitoring in the T_0_ and T_1_transgenic MR219 seeds

**Figure 3:**
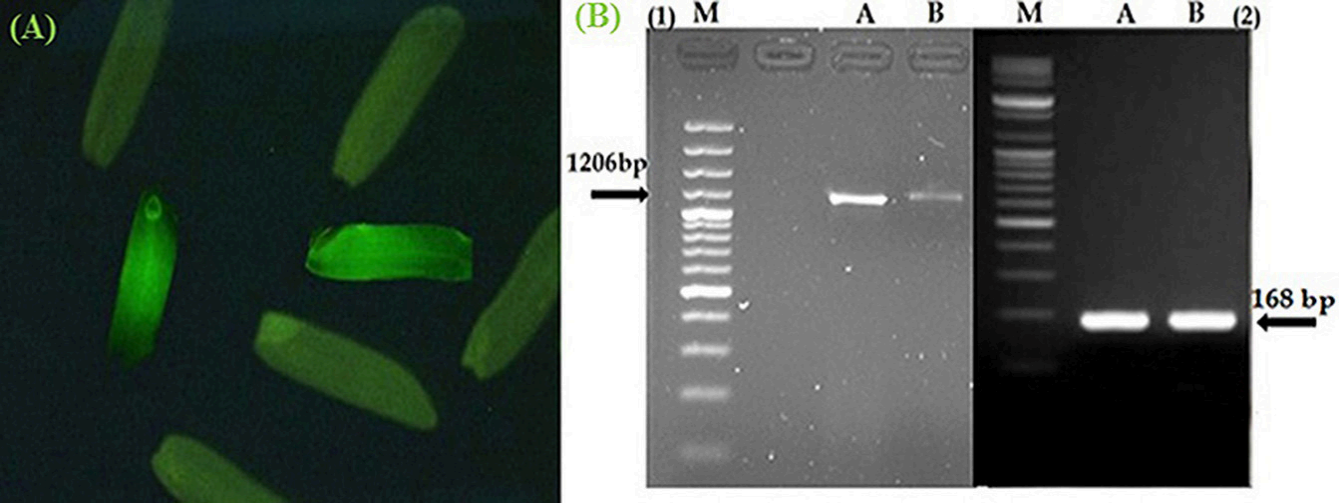
**Confirmation of *Pikh* gene expression in the transgenic and control rice plants. (A)** The expression of *gfp* flanked our gene of interest in the T_1_ seeds. **(B)** Expression pattern of the *Pikh* gene after transgenic rice plant inoculation. (1), RT-PCR analysis was performed using *Pikh*-specific primers using RNA isolated from the inoculated transgenic and wild-type rice plants (A, B, respectively); (2), *18S rRNA* as an internal control.

The seeds harvested from the T_0_ and T_1_ generations were monitored under a microscope to determine the *gfp* gene expression levels. The Figure 3A shows the *gfp* expression among the harvested seeds. Thus, the seeds expressing the *gfp* as observed under the microscope were selected (13.7 and 23% seeds from T_0_ and T_1_) and used for the next generation and different analysis.

### Analyzing the differential expression of the *Pikh* gene in transgenic rice plants (T_1_)

The semi-quantitative PCR analysis showed that the *Pikh* allele was originally presented in the susceptible line (MR219); however, the *Pikh* gene expression levels were generally higher in the inoculated transgenic plants than the (untransformed) control MR219 plants. The *Pikh* gene expression levels after 31 h of inoculation in the transgenic plants were higher compared with the treated control plants (Figure 3B). Real-time quantitative PCR confirmed the results obtained from semi-quantitative PCR. The relative transcript abundance of *Pikh* in the inoculated transgenic plants was higher (expression ratio = 128) compared with the treated wild-type plants (Expression ratio = 4). The differences among the inoculated control and transgenic rice plants were statistically significant (*P* < 0.01). The *Pikh* gene was up-regulated in the inoculated transgenic rice plants by *M. oryzae* (compared with the inoculated wild-type plants) with a *pH* = 0.000.

### Amino acid analysis of the transgenic rice plants (T_1_)

The peak area from the HPLC chromatogram of certain amino acids significantly increased through *Pikh* gene expression in the transgenic plants. The quantity of leucine amino acid differed significantly between the experimental samples with a probability of (*P* < *0.05*). The results of this experiment show that the quantity of leucine amino acid differed significantly in the transgenic plants (47.865 mg g^−1^ DW) compared with wild-type rice plants (17.131 mg g^−1^ DW) using Student's *t*-test (Supplementary Table 2).

### Predicted features for the protein encoded by *Pikh* using bioinformatic tools

**Figure 4:**
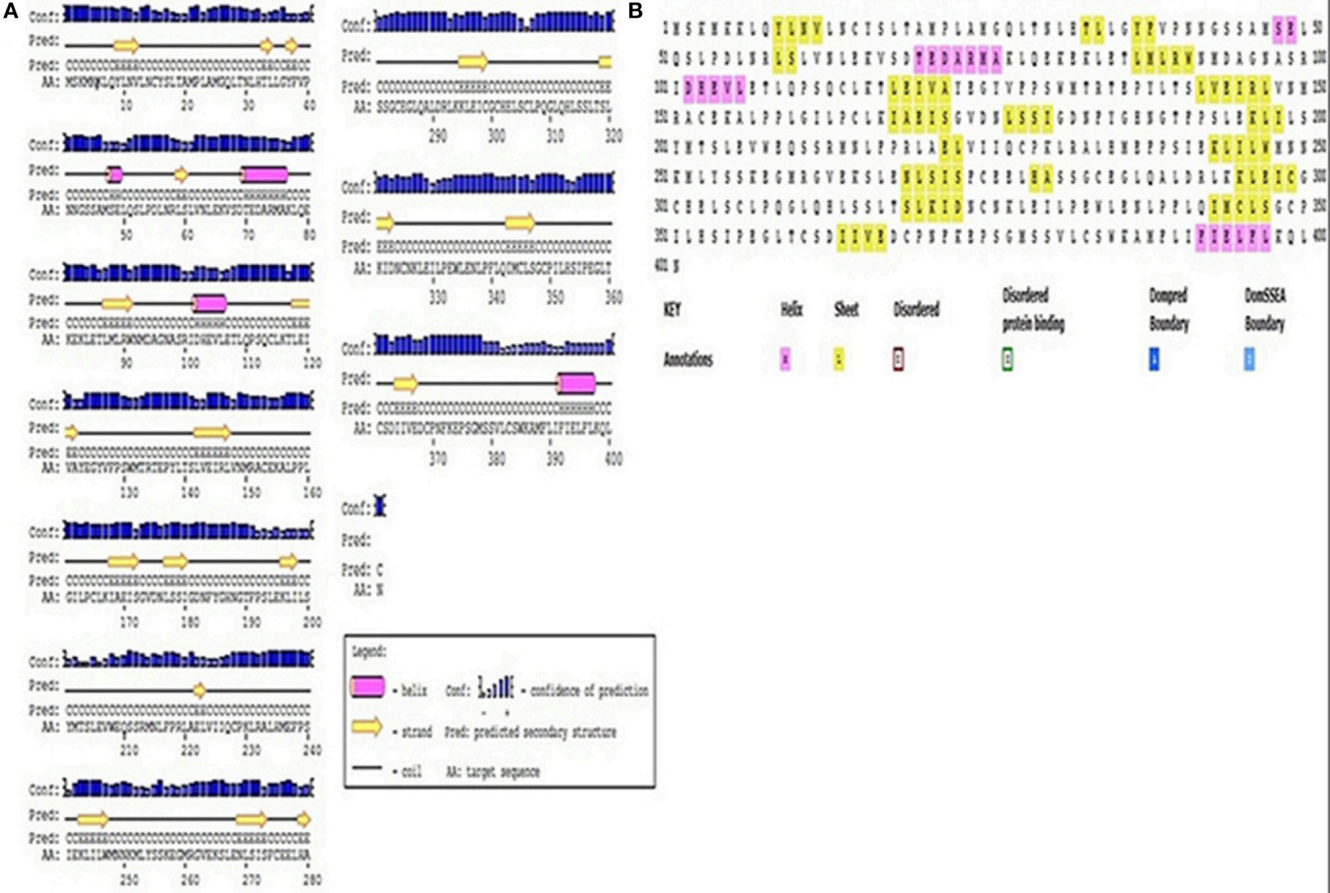
**Secondary structure map of the protein encoded by *Pikh*. (A)** Using PsiPred and **(B)** ITASSER software.

Understanding the hydrophobicity and hydrophilicity is necessary to predict the secondary structure and division of the domain function based on the hypothesis that both hydrophilicity and hydrophobicity are linked to the amino acid score. Based on the quantity of hydrophilic amino acid residues (9.7% of amino acids are represented by leucine), we predict that the *Pikh* gene encodes a hydrophilic protein (Supplementary Figure 4). The prediction results for the secondary structure of the protein encoded by *Pikh* show that the secondary structure consists of 18 sheets, 4 helixes, and 21 coils (Figures 4A,B).

**Figure 5:**
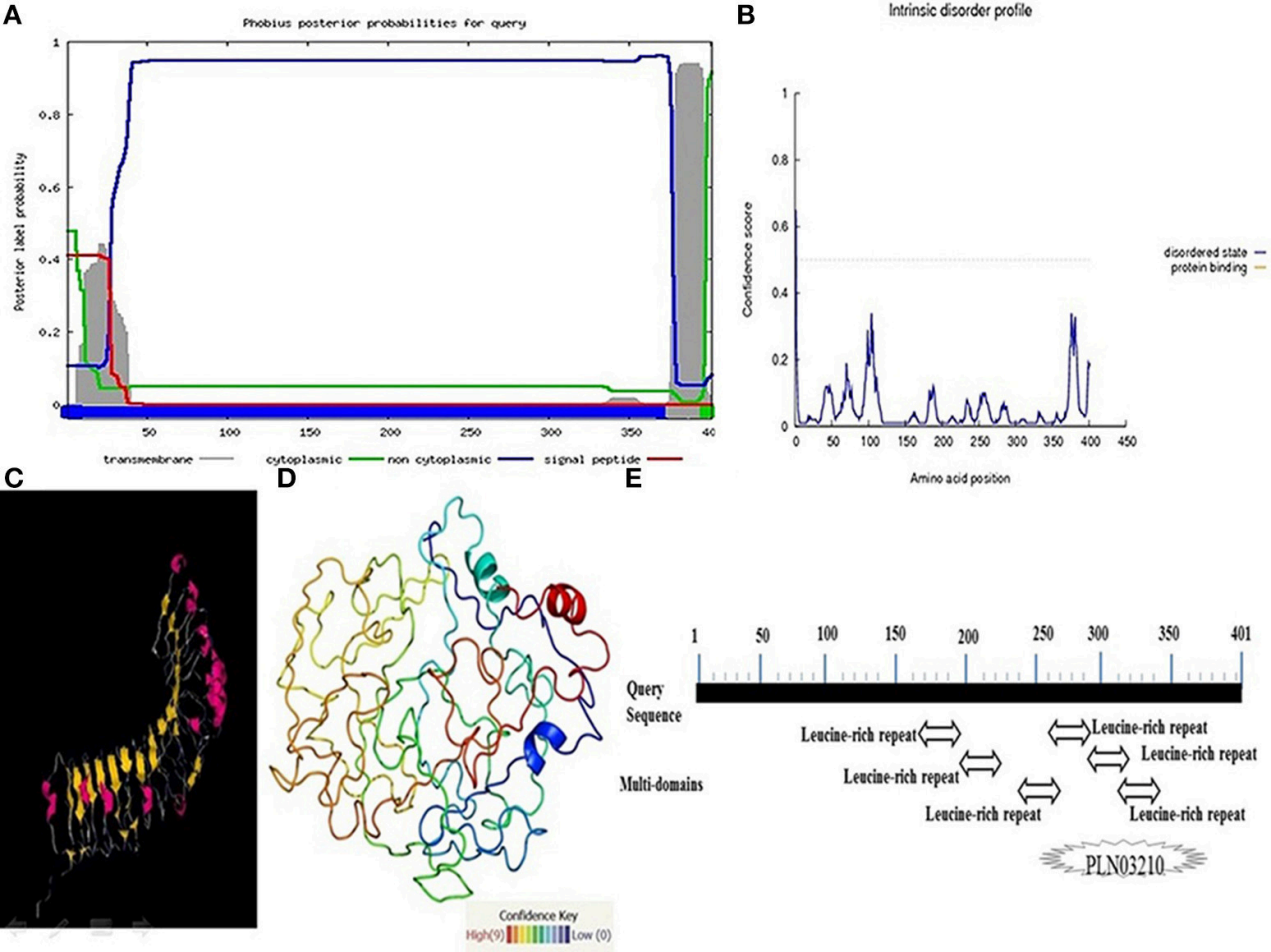
**More investigations on the protein encoded by the *Pikh* gene. (A)** Subcellular localization of the protein encoded by *Pikh*. **(B)** Disordered regions of the protein encoded by *Pikh* predicted using DISOPRED 2. **(C,D)** 3D structures of proteins encoded by the *Pikh* gene predicted by the Phyre2 and ITASSER programs. **(E)** Functional domains predicted at protein encoded by *Pikh* gene.

The *Pikh* gene isolated from the PH9 variety contains 1206 bp coding regions that code for a 401-amino acid protein with a predicted molecular mass of 45.27 kDa. Predicting the subcellular localization of the protein with PSORT II, MEMSAT-SV, Cell-Ploc, and BacelLO demonstrated that the amino acid sequence from 1 to 376 is a non-cytoplasmic region, the sequence from 377 to 397 is a transmembrane region, and the sequence from 398 to 401 is a cytoplasmic region (Figure 5A). In addition, no disordered regions were identified in the protein sequence. Amino acids in the sequence were considered disordered when the blue line was above the gray dashed line, which indicates a confidence score higher than 0.5 (Figure 5B).

We used the Phyre2 (Protein Homology Analogy Recognition Engine) server and ITASSER program to predict the 3D structure of the protein encoded by the *Pikh* gene. Prediction of 3D structures of protein compounds is used to obtain faster information about the structure-activities relationship of known protein compounds (Gruber et al., 2008). A Phyre2 output model was generated based on the template galactose-binding domain-like (Figures 5C,D). We could also identify 6 LRR domains at Pikh protein sequence depending on the algorithm used at NCBI, while there are not detected any NB-ARC domain (Figure 5E).

### Quantification of the fungal population in the inoculated transgenic and untransformed plants using real time-quantitative PCR

Significant variation was detected in the *M. oryzae* content of the inoculated control (untransformed) and transgenic plants in all samples collected 31, 48, and 72 h after inoculation. The expected PCR amplicon size was 330 bp for the *28S rDNA* of the blast fungus (Supplementary Figure 5A). The standard curve for the target gene is shown in Supplementary Figure 5B. The standard curve was constructed using the plot of the *28S rDNA* gene copy numbers for *M. oryzae* against its *Cq*-value. The standard curves showed high correlation coefficients at *R*^2^ = 0.996, which indicate that the *Cq*-value was proportional to the *28S rDNA* gene copy numbers for the blast fungus. From the liner regression slope of −3.353, the amplification efficiency was 98.7% for *M. oryzae*. A good reaction should produce an amplification efficiency between 90 and 110%, and the *R*^2^-value should be > or = 0.985. In this study, the amplification efficiency of 98.7% and the *R*^2^-value of 0.996 for the *M. oryzae* standard curves were within the acceptable range. The amplification curve for *M. oryzae* (Supplementary Figure 5C) was constructed by plotting the cycle numbers against the fluorescence signals (RFU, relative fluorescence units). No fluorescence signals were detected from the NTC. Supplementary Figure 5D shows the melting curve for the blast fungus. From the results, a melting temperature of 86.5 was detected suggesting that the set of primers was specific for identifying and estimating *M. oryzae*.

**Figure 6:**
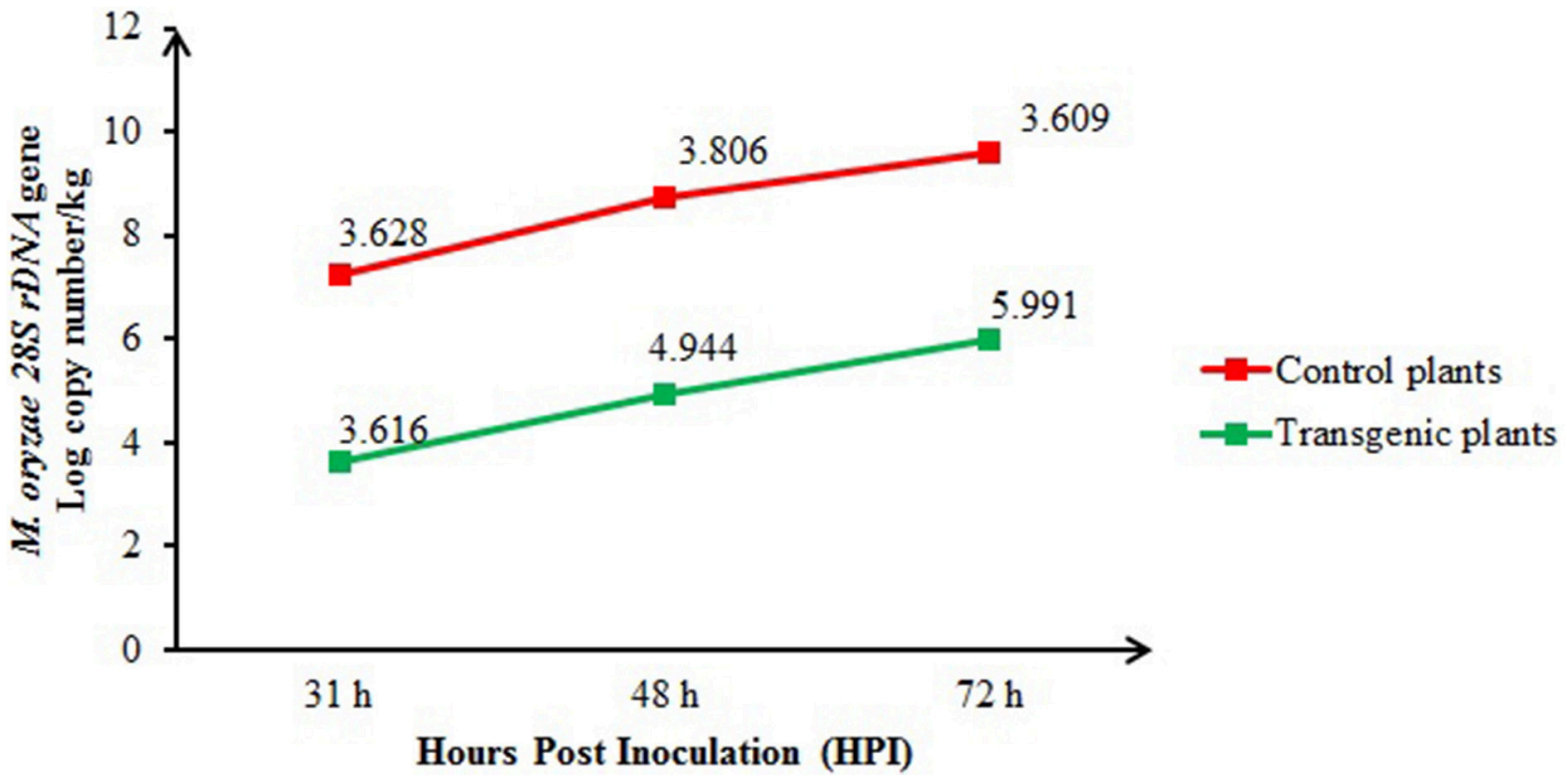
**Comparison of the *M. oryzae* populations among the inoculated transgenic (T_1_) and control plants at 31, 48, and 72 h after inoculation and quantified using real-time quantitative PCR**. The bars represent the means of three biological samples from each time group.

The real-time quantitative PCR quantification results for the *M. oryzae* population of inoculated control (non-transgenic) and transgenic plants at 31, 48, and 72 h after inoculation are shown in Figure 6. At 48 and 72 h after inoculation, the fungal population in the control plants was significantly (*P* < 0.05) higher [4.944 and 5.991 log *28S rDNA* gene copy number/per kg Fresh weight (FW) of plant, respectively] than in the transgenic plants (3.806 and 3.609 log *28S rDNA* gene copy number/per kg FW of plant, respectively). However, at 31 h of inoculation, the blast fungus populations in the transgenic plants (3.616 log *28S rDNA* gene copy number/per kg FW of plant) did not differ significantly from the control plants (3.628 log *28S rDNA* gene copy number/per kg FW of plant).

### Resistance analysis

The inheritance of the *Pikh* gene in the selfed T_1_ generation was analyzed for the phenotype of blast disease resistance to the *M. oryzae* pathotype P7.2. The BLT, BLD and percentages of DLA improved amongst the transgenic plants (3, 1, and 5%, respectively) compared with the control plants (4, 7, and 50%, respectively).

## Discussion

*Magnaporthe oryzae* is a rice blast fungus and a severe pathogen that jeopardizes the second important food-security crop in the world. *M. oryzae* P7.2 has been reported as the most destructive pathotype (Ashkani, 2011). The MR219 rice variety with valuable characteristics (high yield, short maturation period, and resistant to bacterial leaf blight disease) was introduced as a plant variety that is susceptible to this pathotype in Malaysia (Azizi et al., 2015a). Based on our previous research, the *Pi9, Pi21*, and *Osw45* blast resistance genes were concurrently expressed in most Malaysian rice varieties and produced blast resistance, and the *Pikh* gene was up-regulated. Thus, they are valuable genes found in the germplasm for development of rice varieties, and they are up-regulated simultaneously with the *Pikh* gene. Among these four genes, only *Pikh* expression was constant in MR219. In addition, *Pikh* gene can be susceptible and resistance alleles in the genome of different varieties, while a single amino acid difference between similar sequences make the alleles susceptible or resistance (Bryan et al., 2000). Therefore, it can be assumed that the allele with same sequence in MR219 is susceptible allele even because of single amino acid difference. The results of this research support our hypothesis that the MR219 resistance against the blast fungal pathotype P7.2 can be improved through *Pikh* gene over-expression in MR219 (Azizi et al., 2015a). For that reason, we did isolate isolate *Pikh* from one of the most resistant varieties (PH9) based on its presence and high levels of expression and to transfer the gene into the MR219 (susceptible) rice variety. *Pikh (Pi54)* was cloned for the first time from Tetep, which is an Indica cultivar in India. Thereafter, it was thoroughly mapped and molecularly characterized (Sharma et al., 2005a,b). The latest study on this gene was carried out to identify a novel allele of *Pikh* using an allele-mining technique. The researchers identified one similar *Pikh* allele from *Oryza sativa* ecotype Bizor-II of 3172 bp as its full length and another allele of 3543 bp as its full length from the *O. sativa* Indica Group ecotype Boha Thulasi Joha (Ramkumar et al., 2010). Other groups have reported partial sequences for the *Pikh* gene from divers rice varieties, which are available in the National Center for Biotechnology Information (NCBI; Accession: KM043886.1, Accession: AY914077.1, etc.). In the current research, we isolated the full length and CDS of the *Pikh* gene from the PH9 variety at 3127 and 1206 bp, which encoded 401 amino acids. Transferring foreign genes to the rice plants through *Agrobacterium tumefaciens* has become a routine approach. *Agrobacterium-*mediated transformation showed certain advantages compared with other gene transformation methods and results in a lower copy number for transgene insertion through an easy, simple and low-cost method (Dai et al., 2001). The pathogenic soil bacteria, *A. tumefaciens*, has been widely used for plant transformation (May et al., 1995; Tzfira et al., 1997; Sun et al., 2006; Bartlett et al., 2008). Two *Agrobacterium* strains, including LBA4404 and GV3101, have frequently been used to transform foreign genes that were induced in binary vectors (Narusaka et al., 2012). In this experiment, transgenic plants were regenerated using an efficient transformation (using *A. tumefaciens* LBA4404) and tissue culture protocol (~50% efficiency).

The molecular delineation in addition to an amino acid analysis of transgenic MR219 plants were shown in this study. The final amino acid analysis results for the transgenic plants compared with the control plants revealed that over-expression of the *Pikh* gene was effective for most amino acid contents because most of the variants feature leucine content. Most of the R genes identified contain a leucine rich repeat (LRR) structural motif, which includes a repeating pattern of 20–30 leucine amino acids (Azizi et al., 2014). This increasing quantity of leucine amino acids in transgenic MR219 plants cannot only be due to the LRR protein encoded by the transformed *Pikh*, but over-expression of the *Pikh* gene may synergistically and/or antagonistically effect the phytohormones, transcription factors, kinase cascades, reactive oxygen species (ROS), etc. The leucine quantity can be affected by certain such activated functions. An important step in plant defense is appropriate perception of stress for a rapid and efficient response. Thereafter, the plants' constitutive defense mechanisms (Andreasson and Ellis, 2010) initiate activation of defense complex signaling cascades (Abuqamar et al., 2009). During abiotic or/and biotic stress, particular ion channels and kinase cascades are stimulated (Fraire-Velázquez et al., 2011); ROS (Laloi et al., 2004), phytohormones, such as ethylene, salicylic acid (SA), jasmonic acid (JA), and abscisic acid (ABA; Spoel and Dong, 2008), gathered and hormonal pathways will be activated, and reprograming of the genetic machinery yields an appropriate defense response and improvement in plant tolerance to reduce the biological damage affected by the stress (Fujita et al., 2006).

Further, LRR-including *R* genes can be sub-classified into two large classes. One class contains the expected gene product, including an N-terminal, extracellular LRR and a membrane anchor, and the other class involves the *R* gene product, which is predicted as cytoplasmic (Azizi et al., 2014). Subcellular localization of resistance protein is one of the main effective factors on their function in the promotion of resistance to the pathogens. The protein encoded by *R* genes has been illustrated to localize in the cytoplasm and the nucleus (Marone et al., 2013). In fact, a large group of R proteins are accumulated in the nucleus in response to the pathogen attacks, which this nuclear localization is essential for the presence of resistance phenotype. Recently, it has been indicated that uncoupling of the resistance response from cell death signaling is connected to the nucleo-cytoplasmic localization of R proteins (Meier and Somers, 2011). Based on our prediction, with the presence of the transmembrane domain, *Pikh* is clearly a LRR type of R protein and not a NBS-LRR as wrongly reported in the previously published manuscripts (Sharma et al., 2012). Our findings expect that *Pikh* encodes a R protein belonging to the LRR class described by Dangl and Jones (2001), which is a hydrophilic protein with a long non-cytoplasmic amino acid sequence (1–376). This functional domain is easily detected in any genuine LRR class of *R* genes. Real-time quantitative PCR is an excellent tool for *M. oryzae* quantification in plants and can be applied for a trustworthy evaluation of fungal pathogenicity (Qi and Yang, 2002). In this study, real-time quantitative PCR for the *M. oryzae* population confirmed the presence of a higher population of fungi on the inoculated control compared with the transgenic plants at 48 and 72 h after inoculation. A larger *M. oryzae* population was previously reported in the leaves of susceptible varieties compared with resistant plants at 48 and 72 h (Azizi et al., 2015a) and 4 and 6 days after blast fungal inoculation (Correa-Victoria and Zeigler, 1993). Hence, we can conclude that the genetically transformed plants can provide a unique condition that is not suitable for more fungal growth. Subsequently, resistance analyses of T_1_ plants confirmed improved resistance in T_1_ plants against the blast fungal pathotype P7.2.

## Conclusion

Amplified gDNA using specific primer showed the presence of *Pikh* gene with 3127 bp length in PH9 resistant rice variety. In addition, an analysis of the *Pikh* gene cloned in a pDrive cloning vector indicates that it contains a 1206 bp coding region that encodes a 401 amino acid protein using homology searches run with the Basic Local Alignment Search Tool (BLAST). This is the first report of isolating and analyzing the sequence of the *Pikh* transcript and its encoded protein in Malaysian rice varieties. Based on this result, an expression clone with the *Pikh* gene and *CaMV 35*S promoter was constructed and successfully introduced into *Agrobacterium*. Transgenic plants were regenerated and produced using an effective transformation and appropriate tissue culture protocol. The amino acid content in the transgenic plants was affected by the *Pikh* transformation compared with the control plants. Further, the most variation was due to the leucine amino acid content. Due to differences in the leucine quantity among the transgenic MR219 plants compared with the control plants, we conclude that this increasing quantity cannot only be due to the protein encoded by the transformed *Pikh* gene. However, expression of the *Pikh* gene may synergistically and/or antagonistically affect the post-reprograming genetic machinery in transgenic plants and vary the leucine. According to the bioinformatic analysis in this study, the *Pikh* gene encodes LRR protein, which is a functional hydrophilic protein with a long non-cytoplasmic amino acid sequence (1–376). Real-time quantitative PCR quantification of the *M. oryzae* population and a resistance analysis of the transgenic plants also verified improved resistance in plants against the blast fungus. From this research, we generated new information on the expression of *Pikh* and its encoded protein, which are involved in improving the resistance mechanism to the blast fungus pathotype P7.2 in the MR219 rice variety. When the blast fungus enters the plant, the plant's immune system is activated. Effector-triggered immunity (ETI) as a branch of the innate immune system in plants is mediated by intracellular receptor molecules comprising nucleotide binding (NB) and LRR domains that individually recognize effectors produced by the specific pathogens. In fact, the binding of receptors and effectors proteins results in the initiation of defense systems and often leads to localized cell death (Chen and Ronald, 2011). The expression of R genes mediated defense response associates with the hypersensitive response, a type of planned cell death, which occurs at the infection site and restricts growth and distribution of pathogen in the plant (Azizi et al., 2014). Activation and over-expression of *Pikh* gene may control the pathogen in the resistant varieties by producing protein that is involved in this innate immune system. Finally, we emphasize that the results of this study will differ with other isolates due to diverse effectors encoded by different isolates and their specific interactions with the rice blast immunity system. Hence, we conclude that the genetically transformed plants can provide unique conditions that are not suitable for more fungal growth.

## Author contributions

PA, MYR, and MS conceived of the study and designed the experiments. PA, MYR, MS, SA, MH, MM, SA, and MFJ performed the experiments, collected the samples and data, analyzed the experimental data, and wrote the manuscript.

## Funding

The authors wish to acknowledge the Long-Term Research Grant Scheme (LRGS), Food Security Project (Grant No. 5525001), Ministry of Higher Education, Malaysia, for the financial support.

### Conflict of interest statement

The authors declare that the research was conducted in the absence of any commercial or financial relationships that could be construed as a potential conflict of interest.

## Footnotes

^1^Genomics and Sequencing Center of University of Rhode Island (URI).
