# Supplementary material for: Over-Expression of the Pikh Gene with a CaMV 35S Promoter Leads to Improved Blast Disease (Magnaporthe oryzae) Tolerance in Rice
Source: Front Plant Sci. 2016 Jun 17;7:773. doi: 10.3389/fpls.2016.00773 (PMC4911359; doi:10.3389/fpls.2016.00773)
Supplement: Supplementary file 1 [file Table1.DOCX]

***Supplementary Material***

**Over-expression of *Pikh* gene with CaMV 35S promoter: Leading to improve blast disease (*Magnaporthe oryzae*) tolerance in rice**

**P. Azizi^1^, M.Y. Rafii^1*^, S.N. A. Abdullah^3^, M. M. Hanafi^3^, M. Maziah^2^, S. Ashkani^1^, M. F. Jahromi^4^, M. Sahebi^3^**

**^*^**Corresponding author: M.Y.Rafii ([mrafii@upm.edu.my](mailto:mrafii@upm.edu.my))

Supplementary Table 1. Amino acid standard concentration for used in the HPLC procedure.

| Amino acid | Concentration (µg/mL) |
| --- | --- |
| Asp | 6.660 |
| Ser | 5.260 |
| Glu | 7.310 |
| Gly | 3.760 |
| His | 7.760 |
| Arg | 8.710 |
| Thr | 5.960 |
| Ala | 4.460 |
| Pro | 5.760 |
| AABA | 1.000 |
| Cys | 6.060 |
| Tyr | 9.060 |
| Val | 5.860 |
| Met | 7.460 |
| Lys | 7.310 |
| Ile | 6.560 |
| Leu | 6.560 |
| Phe | 8.260 |

Supplementary Table 2. Amino acid profile for the wild-type and transgenic MR219 (mg/g^-1^) plants (T_1_).

| Amino acid | Transgenic  (± SE) | wild-type  (± SE) | Significant |
| --- | --- | --- | --- |
| Aspartic acid | 10.569±0.07 | 10.4±0.05 | NS |
| Serine | 10.526±0.001 | 15.662±0.001 | ** |
| Glutamic acid | 21.383±0.04 | 21.096±0.01 | * |
| Glycine | 1.545±0.001 | 1.555±0.002 | NS |
| Histidine | 28.137±0.001 | 28.789±0.005 | ** |
| Arginine | 13.184±0.001 | 12.905±0.006 | ** |
| Threonine | 19.202±0.001 | 19.04±0.01 | NS |
| Alanine | 1.419±0.001 | 1.419±0.0005 | NS |
| Proline | 10.36±0.01 | 10.498±0.001 | NS |
| Cysteine | 1.011±0.001 | 0.111±0.004 | ** |
| Tyrosine | 4.384±0.002 | 4.542±0.009 | NS |
| Valine | 12.259±0.0005 | 12.037±0.001 | NS |
| Methionine | 2.328±0.001 | 2.241±0.001 | NS |
| Lysine | 7.764±0.004 | 7.899±0.006 | NS |
| Isoleucine | 9.362±0.009 | 8.826±0.03 | ** |
| Leucine | 47.865±0.002 | 17.131±0.6 | ** |
| Phenylalanine | 10.208±0.002 | 9.727±0.003 | ** |

NS: Not significant; * significant at *P*< 0.05 and ** significant at *P*< 0.01.
