# Supplementary figures and images for: Over-Expression of the Pikh Gene with a CaMV 35S Promoter Leads to Improved Blast Disease (Magnaporthe oryzae) Tolerance in Rice
Source: Front Plant Sci. 2016 Jun 17;7:773. doi: 10.3389/fpls.2016.00773 (PMC4911359; doi:10.3389/fpls.2016.00773)

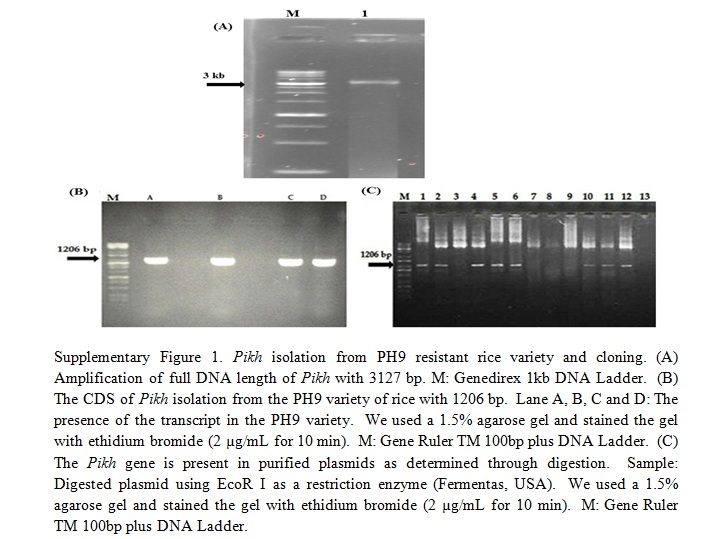

Supplement: Supplementary file 2 [file Image1.JPEG]

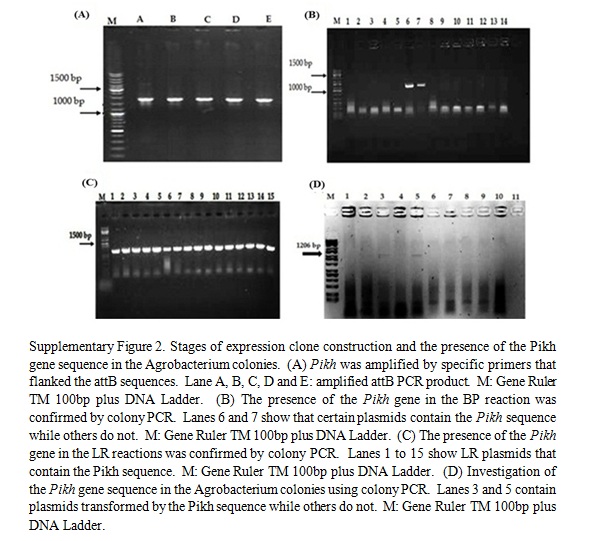

Supplement: Supplementary file 3 [file Image2.JPEG]

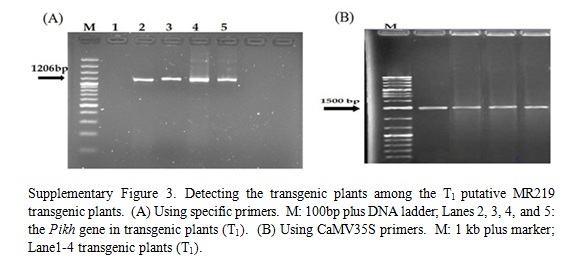

Supplement: Supplementary file 4 [file Image3.JPEG]

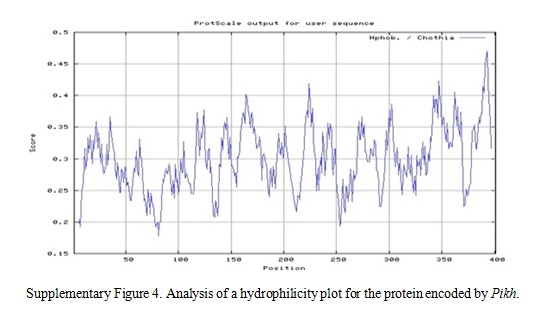

Supplement: Supplementary file 5 [file Image4.JPEG]

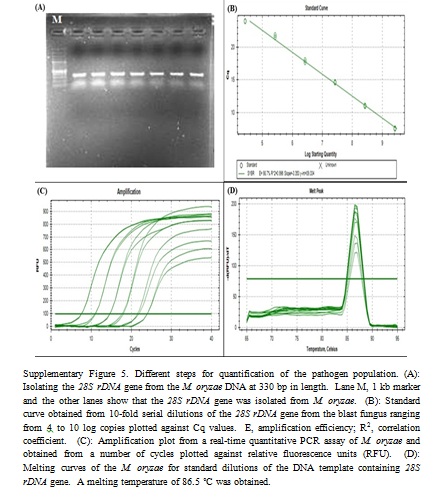

Supplement: Supplementary file 6 [file Image5.JPEG]
